# Transanal endoscopic microsurgery (TEM) for rectal GI stromal tumour

**DOI:** 10.1007/s00464-026-12628-5

**Published:** 2026-02-04

**Authors:** Alberto Arezzo, Giovanni Distefano, Carlo Alberto Ammirati, Michele Barbiero, Simone Arolfo, Mario Morino

**Affiliations:** Department of Surgical Sciences, University of Turin, Corso Dogliotti 14, 10126 Turin, Italy

**Keywords:** GIST Gastro intestinal stroma tumour, Transanal endoscopic microsurgery (TEM), Organ preservation, Salvage surgery

## Abstract

**Background:**

Rectal gastrointestinal stromal tumours (GIST) are uncommon and technically challenging to treat due to the confined pelvic anatomy and proximity to the anal sphincter complex. The oncological goal of surgery is R0 resection, but radical procedures often compromise function. Transanal endoscopic microsurgery (TEM/TEO) provides stable exposure and precise full-thickness excision, offering the possibility of organ preservation. The role of tumour size and neoadjuvant imatinib in enabling local excision remains under investigation.

**Methods:**

We conducted a retrospective single-centre study of all consecutive patients undergoing TEM/TEO for rectal GIST between January 2007 and May 2023. Clinicopathological data, operative outcomes, postoperative course, and follow-up were analysed. Disease-free survival (DFS) and overall survival (OS) were estimated with the Kaplan–Meier method.

**Results:**

Thirteen patients were included. Median age was 55 years (IQR 48–69), 69.2% were male, and median BMI was 26.1 kg/m^2^ (IQR 22.4–28.0). The median tumour distance from the anal verge was 6.0 cm (IQR 4.0–7.0), and the median pathological size was 5.0 cm (IQR 5.0–7.0). Two patients (15.4%) received neoadjuvant imatinib. Spinal anaesthesia was used in 69.2% of cases, with a median operative time of 80 min (IQR 60–110 min). Peritoneal opening occurred in one case (7.7%), which was repaired transanally. No conversions were required. Median hospital stay was 4 days (IQR 2–6), with no recorded postoperative complications; one patient (7.7%) required salvage reintervention. R0 resection was achieved in 92.3%. At a median follow-up of 60 months, two patients (15.4%) developed local recurrence and one patient (7.7%) died. The 12-month Kaplan–Meier estimates were 92.3% for DFS and 100.0% for OS.

**Conclusion:**

TEM/TEO achieved a high rate of R0 resection with organ preservation in this small single-centre series of rectal GIST. Tumour size alone should not be considered an absolute contraindication when en-bloc excision without rupture and negative margins are technically achievable. Neoadjuvant imatinib may facilitate local excision in selected borderline cases, but our experience is limited and larger multicentre registries are required to better define selection criteria and long-term outcomes.

**Supplementary Information:**

The online version contains supplementary material available at 10.1007/s00464-026-12628-5.

Gastrointestinal stromal tumours (GIST) are the most common mesenchymal neoplasms of the gastrointestinal tract, arising predominantly in the stomach and small intestine. Rectal localisation, however, is rare, accounting for fewer than 5% of cases [1]. Owing to the anatomical confines of the pelvis and the proximity of the rectum to the anal sphincter complex, surgical management of rectal GIST presents unique technical and functional challenges [2].

GISTs are KIT/DOG-1–driven tumours for which the primary surgical goal is complete resection of the primary tumour with microscopically negative margins (R0). Unlike adenocarcinoma, lymphatic spread is exceptional, and routine lymphadenectomy is unnecessary. Historically, radical resections such as abdominoperineal excision or low anterior resection were frequently undertaken to ensure negative margins, but these approaches often carry significant morbidity, the risk of permanent stoma, and adverse impacts on quality of life [3, 4].

The advent of minimally invasive transanal platforms, including transanal endoscopic microsurgery (TEM) and transanal endoscopic operation (TEO), has expanded the possibilities for organ-preserving surgery [5]. These systems provide stable insufflation, magnified vision, and access to the mid and upper rectum, enabling precise full-thickness excision and secure closure. Initial reports have demonstrated their feasibility for benign and early malignant lesions, and an increasing body of evidence supports their use in selected rectal GISTs [6–8]. Recent international survey data confirm that local excision remains widely adopted for rectal neoplasia, although indications, follow-up pathways, and platform selection vary significantly across centres, highlighting the need for standardised organ-preserving protocols [9].

Tumour size has traditionally been regarded as a significant determinant of surgical strategy, with lesions larger than 5 cm often considered unsuitable for local excision. Recent series, however, challenge this dogma, showing that transanal resection can achieve oncological outcomes comparable to those of radical surgery, even for larger tumours, while preserving continence and avoiding stoma formation [10, 11]. Additionally, the availability of imatinib, a tyrosine kinase inhibitor that targets KIT and PDGFRA mutations, has significantly transformed the therapeutic landscape. Used in the neoadjuvant setting, imatinib can reduce tumour volume, restore surgical planes, and facilitate complete local excision in cases that might otherwise necessitate mutilating surgery [12, 13]. Comparable organ-preserving strategies have also shown favourable outcomes in other rectal subepithelial tumours such as neuroendocrine lesions, where multiple minimally invasive resection techniques achieve high rates of complete excision with minimal morbidity [14].

Despite these advances, data on transanal endoscopic resection of rectal GIST remain limited, particularly in Western centres. The role of neoadjuvant imatinib, the validity of the 5-cm size threshold, and the long-term implications of organ-preserving strategies remain incompletely defined [8].

The present study reports our institutional experience of rectal GIST managed with TEM/TEO between 2007 and 2023. We aimed to evaluate peri-operative safety, pathological outcomes, and oncological results, with particular emphasis on the feasibility of R0 resection, the role of neoadjuvant imatinib, and the potential for universal organ preservation in this challenging setting.

## Materials and methods

### Study design and setting

This was a retrospective, single-centre cohort study of patients with rectal GIST treated by transanal endoscopic microsurgery (TEM) between January 2007 and May 2023 at the Department of General Surgery, University of Turin. All data were extracted from a prospectively maintained database. Given the accrual period, we acknowledge potential temporal heterogeneity in imaging protocols, platform evolution (TEM/TEO), and peri-operative pathways. The study was conducted in accordance with the Declaration of Helsinki and approved by the local institutional review board.

### Patient selection

Eligible patients had histologically and immunohistochemically confirmed rectal GIST managed with TEM. Patients with metastatic disease at presentation, local recurrence after prior rectal surgery, concomitant malignancies, or incomplete records were excluded.

### Data collection

The following variables were retrieved:

- Clinical characteristics: age, sex, body mass index (BMI), distance of tumour from the anal verge, tumour size, and anatomical location.
- Peri-operative management: use of neoadjuvant or adjuvant imatinib.
- Operative details: anaesthetic technique (spinal or general), operative time, intra-operative complications, peritoneal opening, and conversions.
- Postoperative outcomes: length of hospital stay, complications (graded by Clavien–Dindo classification), and re-interventions within 30 days.
- Pathological findings: resection margin status (R0 or R1), definitive tumour size, and immunohistochemical profile.
- Oncological outcomes: local recurrence, distant recurrence, disease-free survival (DFS), and overall survival (OS).

Follow-up was performed at regular intervals by outpatient review, endoscopy, and cross-sectional imaging according to international guidance (NCCN/ESMO), every six months for the first two years and annually thereafter [4, 15].

### Statistical analysis

Continuous variables are reported as median and interquartile range (IQR) or mean and standard deviation (SD), as appropriate. Categorical variables are expressed as frequencies and percentages. Comparisons between subgroups were performed using the Student’s *t* test or Mann–Whitney U test for continuous variables, and the *χ*^2^ test or Fisher’s exact test for categorical variables.

**Fig. 1:**
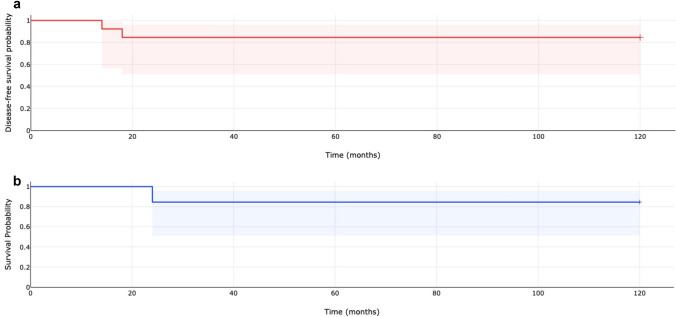
Kaplan–Meier curves showing **a** disease-free survival (DFS) and **b** overall survival (OS) in a cohort of 13 patients undergoing transanal endoscopic microsurgery/operation (TEM/TEO) for rectal GIST

Time-to-event outcomes were analysed using the Kaplan–Meier method (Fig. 1). DFS was defined as the interval between surgery and local or distant recurrence, or death related to the disease. OS was defined as the time from surgery to death from any cause. Patients without an event were censored at the date of last follow-up. Survival curves were compared with the log-rank test.

All statistical tests were two-sided, and a *p*-value < 0.05 was considered statistically significant. Analyses were performed using Python (pandas, matplotlib) and confirmed by standard statistical software.

## Results

### Patient characteristics

A total of 13 consecutive patients underwent transanal endoscopic surgery for rectal GIST at our institution. The median age was 55 years (48–69), with a predominance of males (9 patients, 69.2%). The median BMI was 26.1 kg/m^2^ (22.4–28.0). Tumours were located at a median distance of 6.0 cm (IQR 4.0–7.0 cm) from the anal verge. The median pathological tumour size was 5.0 cm (IQR 5.0–7.0 cm). T tumour size was ≥ 5 cm in 10 of 13 patients (76.9%). Distribution was predominantly anterior (7/13, 53.8%), followed by posterior (3/13, 23.1%) and lateral sites (3/13, 23.1%). Neoadjuvant imatinib was administered in 2 patients (15.4%) when clivages with surrounding organs (e.g. prostate) were unclear and was paused for surgery when the lesion stopped reducing in size on MRI. All patients in this cohort were treated after routine availability of imatinib.

### Operative outcomes

All procedures were completed transanally without conversion. Spinal anaesthesia was used in 9 patients (69.2%), whereas general anaesthesia was required in 4 patients (30.8%). The median operative time was 80 min (IQR 60–110 min). A peritoneal opening occurred in 1 patient (7.7%) and was managed successfully with primary closure.

### Postoperative outcomes

The median hospital stay was 4 days (IQR 2–6). No postoperative complications were recorded.

### Pathological findings

R0 resection was achieved in 12 patients (92.3%), while R1 margins were identified in 1 patient (7.7%) who did not undergo neoadjuvant Imatinib therapy. Histopathology confirmed GIST in all cases, with immunohistochemistry (KIT/DOG-1) positive where performed.

### Oncological outcomes

At a median DFS follow-up of 60 months, there was 1 documented DFS event and 2 local recurrences (15.4%). At a median OS follow-up of 82 months, there was 1 unrelated death (7.7%).

The 12-month Kaplan–Meier estimates were 92.3% for DFS and 100.0% for OS. Table 1 provides a comprehensive summary of baseline, operative, postoperative, pathological, and oncological outcomes.

**Table 1:** Baseline, operative, postoperative, and oncological outcomes

Variable	Result
Age, years	55 (IQR, 48–69)
Gender	
Male	9 (69.2%)
Female	4 (30.8%)
BMI, kg/m^2^	26.1 (IQR 22.4–28.0)
Distance from anal verge, cm	6.0 (4.0–7.0)
Tumour size (pathology), cm	5.0 (5.0–7.0)
Location	
Anterior	7 (53.8%)
Posterior	3 (23.1%)
Lateral	3 (23.1%)
Neoadjuvant imatinib	2 (15.4%)
Anaesthesia	
General	4 (30.8%)
Spinal	9 (69.2%)
Operation time, min	80 (IQR 60–110)
Peritoneal opening	1 (7.7%)
Conversion to laparoscopy	0 (0.0%)
Length of stay, days	4 (IQR 2–6 days)
Postoperative complications	0 (0.0%)
Resection margins	
R0 resection	12 (92.3%)
R1 resection	1 (7.7%)
Local recurrence	2 (15.4%)

## Discussion

A clear oncological principle guides the surgical management of rectal GIST: the aim of surgery is complete local excision of the primary tumour with microscopically negative margins (R0 resection). Routine lymphadenectomy is not indicated because nodal spread is rare [16], so the main intra-operative challenge is to achieve R0 while minimising functional impairment. In our consecutive series, TEM/TEO consistently delivered precise full-thickness excision and secure closure, resulting in an R0 rate of 92.3%, low peri-operative morbidity, and no conversions—thereby meeting the core oncological objective without compromising function. These results reinforce the concept that oncological radicality and functional preservation are not mutually exclusive goals when the enabling platform and case selection are appropriate [5, 17].

Selection for local excision in rectal GIST should be grounded in GIST-specific principles—en-bloc excision with intact capsule, avoidance of tumour rupture, and feasibility of achieving negative margins—rather than extrapolations from rectal adenocarcinoma pathways.

Historically, a 5 cm threshold has often been regarded as a practical upper limit for local excision in rectal GIST, primarily due to concerns regarding margin adequacy, specimen fragmentation, and the potential for increased recurrence risk when dissection is challenging or the capsule is breached. Nevertheless, our experience, together with contemporary evidence, indicates that 5 cm is not an absolute barrier. In a single-centre comparative study of rectal GIST > 5 cm, transanal endoscopic local resection achieved no significant difference in 5-year DFS compared with transabdominal radical resection (94.1% vs. 100%; *p* = 0.405), while conferring secondary advantages including shorter operative time, reduced length of stay, and higher anal-preservation rates. In practice, these data argue against using tumour size alone to deny a transanal approach. Instead, they support individualised decision-making that integrates tumour biology (driver mutations, mitotic activity where available), location relative to the sphincter complex and peritoneal reflection, radiological resectability, and anticipated response to systemic therapy [18].

Recent comparative datasets further support an individualised organ-preserving strategy when an R0 excision can be achieved. A propensity-score matched analysis of the National Cancer Database found no oncologic disadvantage for local excision compared with radical resection in appropriately selected rectal GIST, while emphasising functional and stoma-avoidance benefits of sphincter preservation [19]. Similarly, a multicentre propensity score-matched analysis of low rectal GIST reported comparable outcomes between local excision and radical resection after matching, reinforcing that anatomical feasibility and tumour biology—rather than size alone—should guide the surgical approach [20]. Finally, systematic efforts to synthesise the evidence base, including a recently published meta-analysis protocol, highlight persistent uncertainty driven by rarity and retrospective designs and support the need for multicentre registries using standardised definitions of R0, tumour rupture, and follow-up [21].

Within this individualised framework, neoadjuvant imatinib is pivotal. By downsizing the tumour and re-establishing tissue planes (“clivages”), imatinib increases the likelihood of a margin-negative local excision and reduces the need for mutilating radical surgery. In our cohort, two patients received neoadjuvant therapy, and both underwent successful R0 local excision with organ preservation. Although our numbers are small, these observations align with prior series and reviews in which neoadjuvant imatinib either enabled TEM in borderline cases or, when margins were microscopically positive, allowed a strategy of repeat local excision or adjuvant targeted therapy with acceptable disease control. Regarding timing, multiple reports suggest a minimum of ~ 6 months with effects plateauing around 9 months—often an optimal window for surgery—underscoring the need for MDT coordination and interval imaging (high-resolution pelvic MRI with endorectal ultrasound) to reassess resectability and plan operative timing [22, 23].

Our series also highlights that organ preservation is achievable in carefully selected patients for TEM/TEO. None of our patients required abdominoperineal resection, stoma formation, or conversion to transabdominal surgery; even a single peritoneal opening was repaired transanally without sequelae. This finding carries significant quality-of-life implications. For a disease where long-term survival is often excellent the avoidance of a permanent stoma and preservation of continence and sexual function are outcomes of substantial value to patients. From a technical standpoint, several aspects likely contributed to universal organ preservation and the high R0 rate in our cohort: disciplined full-thickness excision with traction/counter-traction to protect the capsule; early identification of the correct dissection plane; systematic specimen orientation to avoid artefactual margin compromise; and dependable closure of the rectal wall defect, even with peritoneal entry. Evidence from gastric GIST further supports this paradigm, as endoscopic full-thickness resection achieves excellent oncological control with reduced length of stay and rapid recovery, reinforcing the suitability of GIST biology for minimally invasive organ-preserving techniques [17]. Importantly, peritoneal opening need not be considered a failure; when recognised promptly and closed primarily, its impact on recovery and oncological safety appears minimal in experienced hands [24].

Despite favourable results, several limitations merit discussion. First, the retrospective design introduces potential selection and information bias (e.g. incomplete recording of blood loss or mitotic index). Second, the cohort is modest, reflecting the rarity of rectal GIST, and thus underpowered for definitive comparisons of anaesthetic strategy or the independent impact of imatinib on margin status and recurrence. Third, the absence of a dedicated database stratifying surgical procedures by tumour site and operative approach limits our ability to precisely estimate how many rectal GISTs were managed through an anterior laparoscopic strategy during the study period. Fourth, while median follow-up was substantial, longer surveillance and standardised risk stratification (including mitotic index and rupture status) would be required for more definitive conclusions on durable disease control. Finally, detailed pathology (e.g. comprehensive mitotic counts and standardised risk stratification) was variably documented, limiting granular modelling [1, 3].

These limitations point to future work. Prospective multicentre registries, using standardised operative reporting and data dictionaries, are needed to capture uniform margin metrics (including deep margin), capsule integrity/rupture, mitotic index, and mutation status, as well as specifics of imatinib exposure (dose, duration, and radiological response), and robust patient-centred functional outcomes. Such infrastructure will refine selection criteria for TEM/TEO across tumour sizes and locations, define anatomical or biological thresholds where R0 probability declines, and quantify the incremental benefit of neoadjuvant therapy in distinct subgroups. Long-term surveillance should prioritise oncological endpoints (local/distant recurrence, disease-specific survival) and validated functional outcomes—metrics that matter to patients and determine treatment satisfaction [25].

Fourth, while median follow-up was substantial, longer surveillance and standardised risk stratification (including mitotic index and rupture status) would be required for more definitive conclusions on durable disease control.

Taken together, our findings support TEM/TEO as a feasible organ-preserving option for selected patients with rectal GIST when en-bloc excision without rupture and negative margins are technically achievable. The classic goals—R0 resection, avoidance of tumour rupture, and organ preservation—are mutually reinforcing when transanal endoscopic surgery is integrated with neoadjuvant imatinib in appropriate patients. Rather than disqualifying patients on an arbitrary size cut-off, a nuanced MDT pathway that considers tumour biology, precise pelvic anatomy, and dynamic response to therapy should govern strategy. In this paradigm, transanal excision is not merely an alternative to radical resection but an enabling platform that, in concert with targeted therapy, delivers oncological adequacy while maximising preservation of anorectal function.

## Supplementary Information

Below is the link to the electronic supplementary material.

Supplementary file1 (MP4 366426 KB)
